# 

**Published:** 1855-05

**Authors:** 


### CONTENTS OF NO. IV., VOL. II.

ORIGINAL COMMUNICATIONS.

Professional Self-Reliance—Valedictory Address of Prof. Sanborn . . . . . . . . 241
Professional Letter—By Epsom Saultz, M. D. . . . . 252
Illustrations of Vicarious Diseased Action—By Prof. Knowles . . . . . . . . . . 258
Excission of Lead Bar—By John Bell, M. D. . . . 261
Operations for Necrosis—By Prof. Hughes . . . 266

DEPARTMENT OF SELECTIONS.

Nature and Treatment of Western Autumnal Diseases —By Prof. Armor . . . . . . 270
On the Use of Cold as an Anæsthetic—By Dr. Arnott . 278
Quickness of Pulse after Fever—By Dr. Stokes . . 282
Importance of Nourishment in Fever—By Dr. Stokes . 285
Solidified Milk . . . . . . . . . 288
Hydroclorate Ammonia in Neuralgia—By Dr. Ebden . 289
Mineral Acids in Nausea, and Vomiting in Pregnancy . 290
Records Boston Medical Society—By R. M. Hodges, M. D. 292
Remedies for Disorders of Stomach—By Geo. Budd, M. D. 294
Inhalations in Pulmonary Diseases . . . . . . 296

EDITORIAL DEPARTMENT.

Hydrophobia . . . . . . . . . . 297
Practice of Drugging Children . . . . . . 302
Charge of Prof. Pancoast . . . . . . . 305
Humiliating . . . . . . . . . . 306
Poem on Rheumatism—By E. S. S. Rouse . . . . 309
A Benevolent Enterprise . . . . . . . 311

REVIEWS AND BIBLIOGRAPHICAL NOTICES.

Error of Position . . . . . . . . 312
Starling Hall . . . . . . . . . 312
Sanitary Commission . . . . . . . . 313
Autibiography of Chas. Caldwell, M. D. . . . 315
Table of Urinary Deposits . . . . . . . 317
Cases Polypus Uterus . . . . . . . . 317
Rand's Medical Chemistry . . . . . . 317
American Medical Association . . . . . . 318
Iowa Medical Society—Notice . . . . . . 319
Dr. Kirtley Ryland . . . . . . . . . 320

